# Isoliquiritigenin Ameliorates High-Fat Diet-Induced Obesity in Mice by Activating Brown Adipose Tissue

**DOI:** 10.3390/ijms26041616

**Published:** 2025-02-14

**Authors:** Le Zhao, Minhao Li, Qingjun Zhu, Xingqiang Fang, Haili Yang, Yongju Zhao

**Affiliations:** Chongqing Key Laboratory of Herbivore Science, College of Animal Science and Technology, Southwest University, Chongqing 400715, China; zhaole0228@163.com (L.Z.); liminhao1222@163.com (M.L.); zzqingjun@163.com (Q.Z.); 15881044561@163.com (X.F.)

**Keywords:** isoliquiritigenin, brown adipose tissue, obesity, thermogenesis, high-fat diet

## Abstract

Brown adipose tissue (BAT) is a critical regulator of non-shivering thermogenesis and energy expenditure, offering significant potential for addressing obesity and associated metabolic disorders. Isoliquiritigenin (ISL), a natural flavonoid, has shown promising therapeutic effects in lipid metabolism-related diseases. This study aimed to explore the effects of ISL on lipid metabolism and obesity using a high-fat-diet (HFD)-induced obesity model in mice. Mice were subjected to an HFD and treated with ISL via gavage. The results demonstrated that ISL treatment significantly reduced HFD-induced weight gain and upregulated the expression of key thermogenic genes, suggesting enhanced BAT activity and thermogenesis. In vitro experiments using C3H10-T1/2 cells further supported these findings, as ISL treatment markedly increased the expression of UCP1 and PPARGC1a, which are critical regulators of thermogenesis. To elucidate the molecular mechanisms underlying ISL’s effects, we conducted a transcriptomic analysis of BAT from ISL-treated mice. Pathway enrichment analysis revealed that differentially expressed genes were predominantly associated with metabolic processes, including the tricarboxylic acid (TCA) cycle, oxidative phosphorylation, and fatty acid degradation. These pathways are integral to energy metabolism and thermogenesis, providing mechanistic insights into ISL’s anti-obesity effects. Additionally, ISL treatment significantly downregulated the expression of NNAT and SGK1, genes implicated in lipid metabolism and energy homeostasis. These findings suggest that ISL modulates BAT function by regulating the expression of these genes, thereby influencing lipid deposition and thermogenic capacity. In summary, this study suggests that ISL treatment has the potential to mitigate HFD-induced obesity by promoting BAT thermogenesis and modulating lipid metabolism. The molecular mechanisms involve the regulation of key metabolic pathways and genes, such as NNAT and SGK1, highlighting ISL’s potential as a therapeutic agent for obesity and related metabolic disorders.

## 1. Introduction

Excess energy in the body is primarily stored in white adipocytes [1]. The overaccumulation of white adipose tissue (WAT) is not only a hallmark of obesity but is also closely linked to various chronic metabolic diseases, such as type 2 diabetes (T2D) [2], hypertension [3], and cardiovascular diseases [4]. Moreover, obesity-induced adipose tissue inflammation, characterized by elevated macrophage infiltration and pro-inflammatory cytokine production (e.g., TNF-α, IL-6), plays a crucial role in the pathogenesis of obesity-associated metabolic disorders. This inflammatory response exacerbates insulin resistance, adipocyte dysfunction, and systemic metabolic derangements, further contributing to the development of diseases such as T2D and cardiovascular diseases [5]. In contrast, brown adipocytes and beige adipocytes can enhance energy metabolism through the production of heat mediated by uncoupling protein 1 (UCP1). These adipocytes contribute to non-shivering thermogenesis, which consumes stored energy and helps regulate energy balance [6]. Therefore, a comprehensive investigation of the regulatory molecules and mechanisms of thermogenesis in brown adipose tissue (BAT) is crucial for developing strategies to combat obesity and its associated metabolic disorders, including T2D, hypertension, and cardiovascular diseases.

Natural products and their phytochemicals have garnered significant interest, particularly for their potential roles in regulating fat metabolism [7]. Researchers have high expectations for the efficacy, safety, and stability of these natural products in this context. Among them, natural flavonoids are a primary focus of research due to their widespread occurrence in plants and their status as one of the most abundant polyphenolic compounds in the human diet [8]. Several in vitro and in vivo studies have now demonstrated significant effects of flavonoids on fat metabolism [9]. Licorice (*Glycyrrhiza glabra*), a pivotal herb in traditional Chinese medicine with over 2000 years of history, contains isoliquiritigenin (ISL), a bioactive chalcone renowned for its potent antioxidant [10], anti-inflammatory [11], antibacterial [12], and anticancer properties [13]. ISL has been demonstrated to effectively inhibit the activation of the NLRP3 inflammasome and reduce inflammation in adipose tissue induced by a high-fat diet (HFD), owing to its potent anti-inflammatory effects [14]. Further studies have found that ISL interrupts the inflammatory signaling between adipocytes and macrophages independently of the inflammasome and reduces fibrosis in adipose tissue by modulating several innate immune responses [15]. Additionally, ISL has been found to affect metabolic disorders related to obesity, such as hepatic steatosis and insulin resistance [16]. ISL can diminish inflammation in adipose tissue and reduce the risk of metabolic syndrome by modifying the gut microbiota composition [17]. Notably, the combination of berberine and ISL synergistically improves adipose inflammation and obesity-induced insulin resistance [18]. ISL also enhances the differentiation potential of adipose-derived stem cells into beige adipocytes by inhibiting the JNK signaling pathway [19]. These findings suggest that ISL holds significant promise as a therapeutic agent against obesity, showing positive results in modulating diseases associated with fat metabolism, although further investigation is needed to fully understand its effects and underlying mechanisms.

Thus, we hypothesized that ISL ameliorates HFD-induced obesity in mice by activating BAT. To test this hypothesis, we aimed to investigate the effects of ISL on thermogenesis in BAT and its potential role in preventing the whitening of BAT induced by HFD. Furthermore, we explored whether ISL regulates adipose deposition through mechanisms involving the expression of *NNAT* and *SGK1*. This study seeks to provide insights into the therapeutic potential of ISL for obesity.

## 2. Results

### 2.1. ISL Treatment Attenuates Development of Obesity Induced by HFD

**Figure 1:**
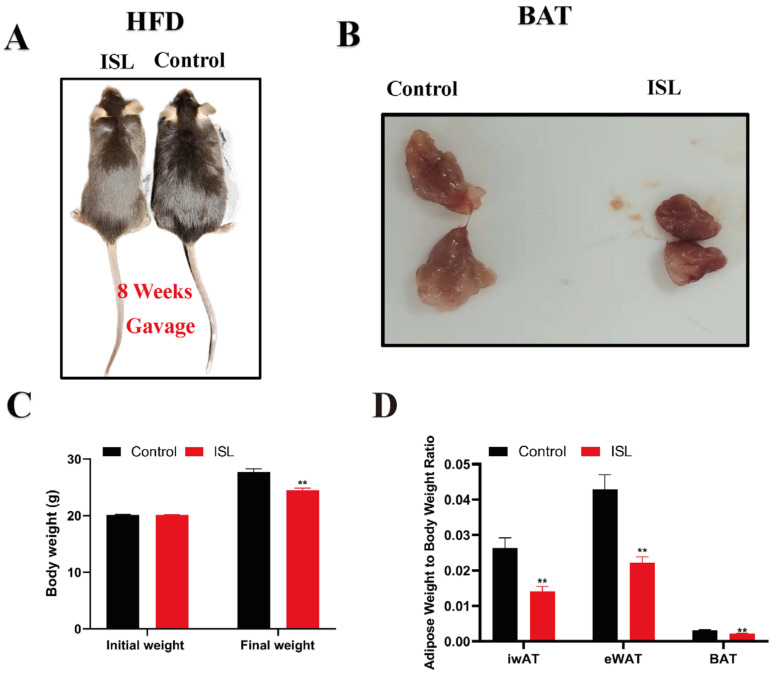
ISL protects against dietary obesity. (**A**) Representative images of mice at the end of the treatment. (**B**) Representative images of the BAT. (**C**) The body weight (*n* = 6). (**D**) Adipose weight-to-body weight ratio (*n* = 6). ** *p* < 0.01.

To assess the effects of ISL on diet-induced obesity, HFD mice were administered 50 mg/kg body weight daily of ISL or its control daily for 8 weeks while maintaining an HFD. Results indicated that the body weight of ISL-treated HFD mice was significantly reduced compared to the control group (*p* < 0.01) (Figure 1A,B). Additionally, the relative weight of each adipose depot, including BAT, iWAT, and eWAT, was significantly reduced in the ISL-treated group compared to the control group (*p* < 0.01) (Figure 1C). These findings suggest that ISL effectively mitigates HFD-induced obesity in mice.

### 2.2. ISL Treatment Inhibits the Expression of Adipogenic Genes

**Figure 2:**
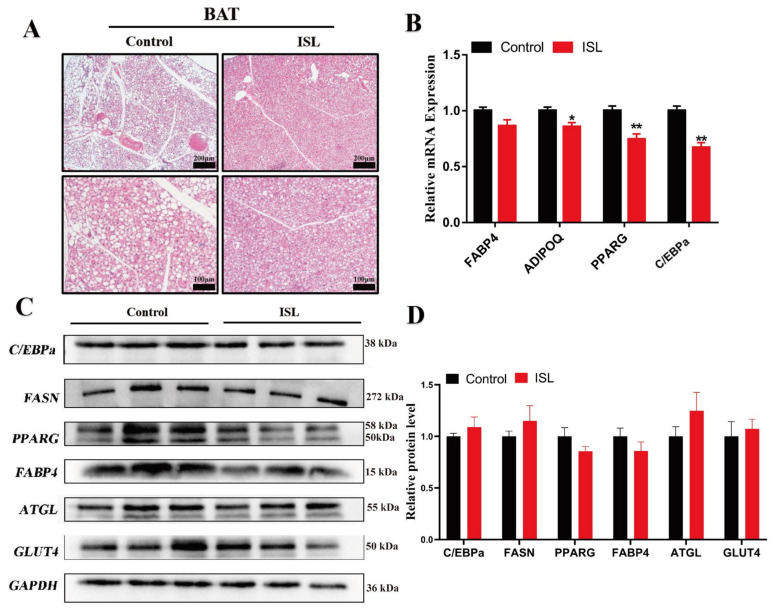
ISL treatment inhibits the expression of adipogenic genes. (**A**) H&E staining of BAT following ISL treatment (*n* = 3). (**B**) The mRNA levels of *FABP4*, *ADIPOQ*, *PPARG*, and *C*/*EBPa* in the BAT (*n* = 3). (**C**,**D**) The protein levels of C/EBPa, FASN, PPARG, FABP4, ATGL, and GLUT4 in BAT (*n* = 3). * *p* < 0.05, ** *p* < 0.01.

ISL demonstrated a significant reduction in body weight among HFD-induced mice. To delve deeper into the mechanisms underlying the weight reduction observed in ISL-treated mice, a histomorphological analysis was conducted, revealing that ISL-treated HFD mice exhibited smaller BAT adipocytes with an increased presence of multilocular small lipid droplets (Figure 2A). A transcriptional analysis indicated a significant downregulation of pro-adipose deposition-related genes, including *ADIPOQ* (*p* < 0.05), *PPARG* (*p* < 0.01), and *C*/*EBPa* (*p* < 0.01), in ISL-treated mice (Figure 2B). Western blot analysis, however, did not reveal any statistically significant differences in the expression levels of C/EBPa (*p* > 0.05), FASN (*p* > 0.05), PPARG (*p* > 0.05), ATGL (*p* > 0.05), and GLUT4 (*p* > 0.05) in the BAT of ISL-treated mice compared to controls (Figure 2C,D).

### 2.3. ISL Treatment Enhanced Mitochondrial Biogenesis and Thermogenesis

**Figure 3:**
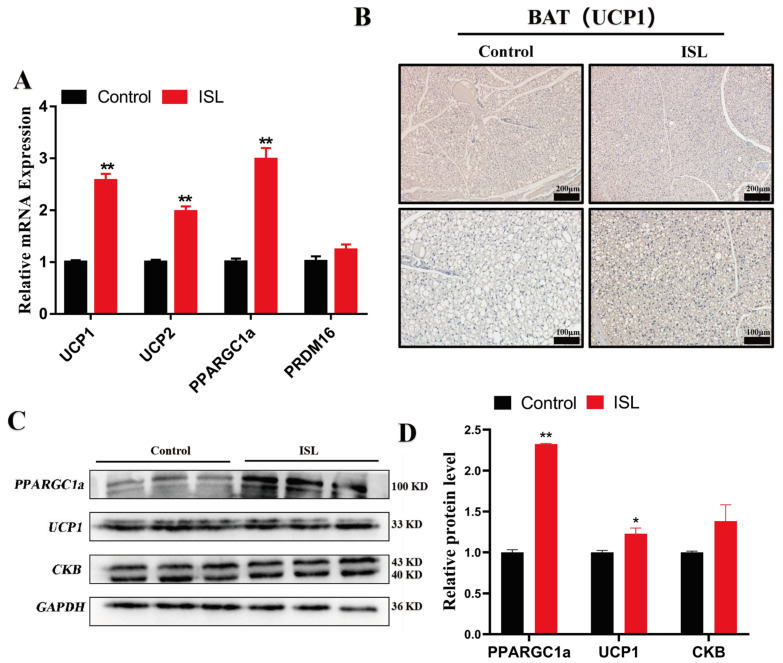
ISL treatment enhanced the expression of mitochondrial biogenesis and thermogenesis-related genes and proteins. (**A**) The mRNA levels of *UCP1*, *UCP2*, *PPARGC1a*, and *PRDM16* in the BAT (*n* = 3). (**B**) Representative UCP1 staining of BAT (*n* = 3). (**C**,**D**) The protein levels of PPARGC1a, UCP1, and CKB in BAT (*n* = 3). * *p* < 0.05, ** *p* < 0.01.

To investigate the underlying mechanisms of ISL-induced weight loss in mice, we conducted a detailed analysis of the expression of genes associated with mitochondrial biogenesis and thermogenesis in the BAT of ISL-treated mice. A transcriptional analysis revealed that ISL treatment significantly upregulated the expression of *UCP1* (*p* < 0.01), *UCP2* (*p* < 0.01), and *PPARGC1a* (*p* < 0.01) (Figure 3A). Furthermore, Western blot analysis corroborated these findings, showing a marked increase in the protein levels of UCP1 (*p* < 0.05) and PPARGC1a (*p* < 0.01) following ISL treatment (Figure 3C,D). These results collectively suggest that ISL enhances mitochondrial biogenesis and thermogenic activity in brown adipose tissue, contributing to its weight loss effects.

### 2.4. ISL Treatment Enhanced Thermogenesis in C3H10-T1/2 Cells

**Figure 4:**
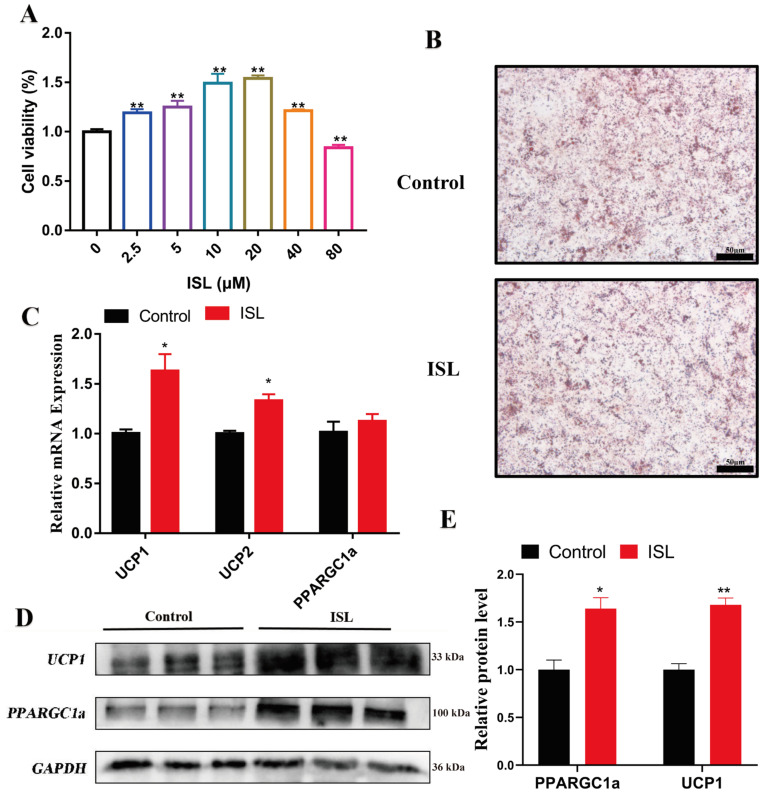
ISL treatment enhanced the expression of thermogenesis-related genes and proteins in C3H10-T1/2 cells. (**A**) CCK-8 assay was performed to evaluate the effect of different concentrations of ISL on the viability of C3H10-T1/2 cells (*n* = 6). (**B**) Oil red O staining. (**C**) The mRNA levels of *UCP1*, *UCP2*, and *PPARGC1a* (*n* = 3). (**D**,**E**) The protein levels of UCP1 and PPARGC1a (*n* = 3). * *p* < 0.05, ** *p* < 0.01.

To further elucidate the capacity of ISL to enhance thermogenesis in brown adipose tissue, we employed C3H10T 1/2 cells as the model system in this study. ISL was incorporated into a lipid-induced differentiation medium, and the expression levels of thermogenesis-related genes were subsequently assessed. The findings indicated that a concentration of 20 μM of ISL elicited a significant and most pronounced promotion of cell viability in C3H10T 1/2 cells (Figure 4A). Following the successful induction of differentiation in C3H10T 1/2 cells at this concentration (Figure 4B), a transcriptional analysis revealed that ISL treatment substantially upregulated the expression of *UCP1* (*p* < 0.05) and *UCP2* (*p* < 0.05) (Figure 4C). Western blot analysis further substantiated these observations, demonstrating that the protein levels of UCP1 (*p* < 0.01) and PPARGC1a (*p* < 0.05) were markedly elevated in ISL-treated cells compared to controls (Figure 4D,E). These data collectively highlight the upregulation of thermogenesis-related genes and proteins in differentiated brown adipocytes following ISL treatment, suggesting its potential role in augmenting energy expenditure and potentially mitigating obesity.

### 2.5. ISL Treatment Alters Transcriptome in BAT

**Figure 5:**
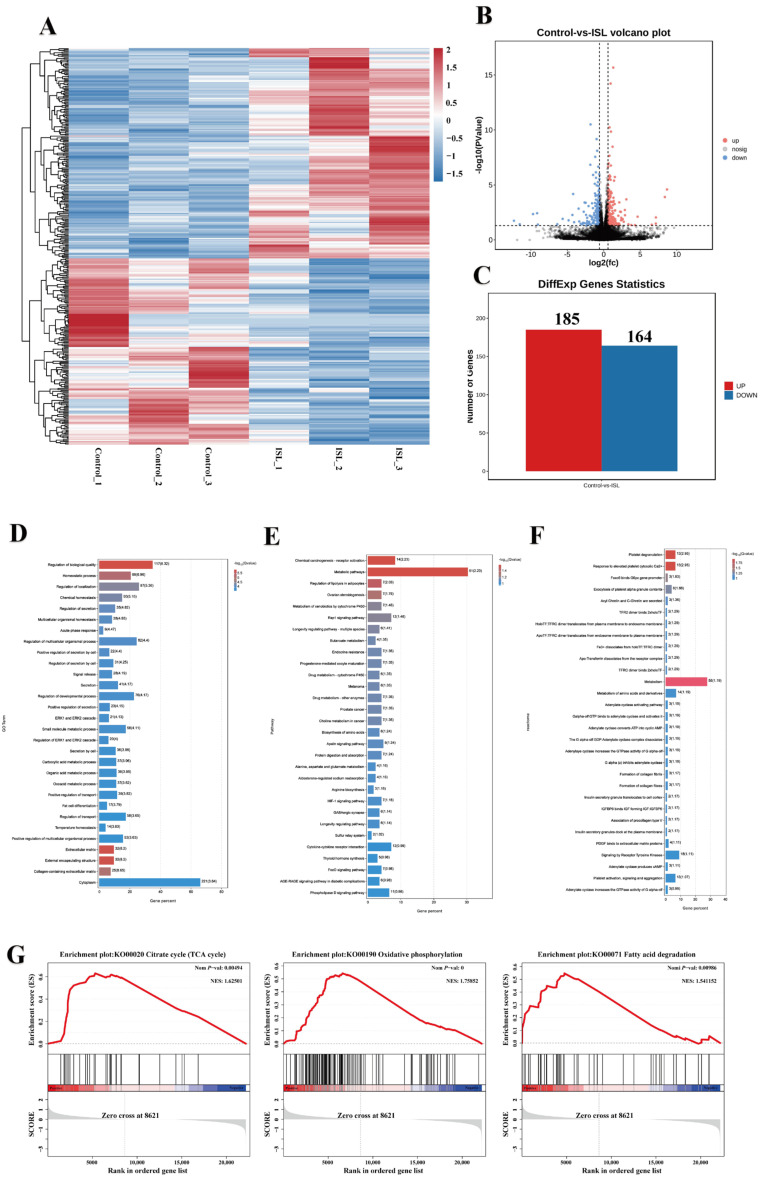
ISL treatment alters transcriptome in BAT. (**A**) Differential gene clustering heat map. (**B**) Differential comparison volcano map. (**C**) Differentially expressed mRNA statistics (upregulated and downregulated). (**D**) GO analysis of DEGs. (**E**) Enrichment barplot for KEGG pathway analysis. (**F**) Enrichment barplot for Reactome pathway analysis. (**G**) GSEA enrichment analysis of citrate cycle (TCA cycle), oxidative phosphorylation, and fatty acid degradation from the control and ISL-treated groups.

To elucidate the molecular mechanisms through which ISL treatment modulates thermogenesis in BAT, we conducted a comprehensive analysis of the gene expression profile in BAT using RNA-seq. Differential expression analysis identified a total of 349 differentially expressed mRNAs between the control and ISL-treated groups, including 185 genes that were upregulated and 164 genes that were downregulated (Figure 5A–C). GO pathway enrichment analysis demonstrated that the DEGs were primarily enriched in categories related to the regulation of biological quality, extracellular matrix organization, homeostatic processes, and collagen-containing extracellular matrix structures. Furthermore, KEGG pathway enrichment analysis indicated that these DEGs were significantly enriched in pathways associated with chemical carcinogenesis—receptor activation, metabolic processes, the regulation of lipolysis in adipocytes, the metabolism of xenobiotics by cytochrome P450, the RAP1 signaling pathway, among other signaling cascades (Figure 5E). Additionally, Reactome pathway enrichment analysis revealed that the DEGs were predominantly associated with signaling pathways, including platelet degranulation, responses to elevated platelet cytosolic Ca^2+^, and Foxo6 binds G6pc gene promoter (Figure 5F). To evaluate the biological effects of ISL treatment on BAT, Gene Set Enrichment Analysis (GSEA) was conducted. The results indicated that three pathways closely associated with brown adipose activity were significantly enriched: the citrate cycle (TCA cycle), oxidative phosphorylation, and fatty acid degradation (Figure 5G). These findings offer valuable insights into the regulatory networks influenced by ISL in brown adipocytes, underscoring its potential impact on thermogenic and metabolic pathways.

**Figure 6:**
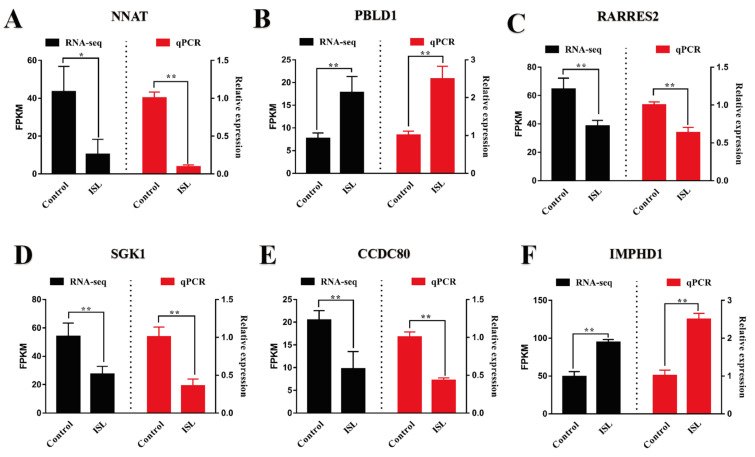
Comparative analysis of gene expression levels in BAT: RNA-Seq versus qRT-PCR results (*n* = 3). (**A**) *NNAT*. (**B**) *PBLD1*. (**C**) *RARRES2*. (**D**) *SGK1*. (**E**) *CCDC80*. (**F**) *IMPHD1*. * *p* < 0.05, ** *p* < 0.01.

qRT-PCR was employed to validate the gene expression alterations induced by ISL treatment in BAT. Six DEGs were selected for further analysis, including *NNAT*, *PBLD1*, *RARRES2*, *SGK1*, *CCDC80*, and *IMPHD1*. The expression levels of these genes were assessed using qRT-PCR (Figure 6A–F). The results from qRT-PCR corroborated the findings from the initial RNA sequencing analysis, confirming the consistency of the transcriptomic changes associated with ISL treatment. These validations underscore the impact of ISL on gene regulation within BAT and support its role in modulating thermogenic processes.

## 3. Discussion

As global obesity rates continue to rise, this condition has emerged as a significant public health challenge, contributing to a range of comorbidities, including diabetes, cardiovascular diseases, and certain types of cancer [20,21,22]. Therefore, identifying safe and effective weight loss interventions has become increasingly important. Recent research underscores the vital role of BAT in energy metabolism and weight management. Unlike white adipose tissue, which primarily stores excess energy, BAT has the unique ability to generate heat through thermogenesis, increasing the basal metabolic rate and enhancing insulin sensitivity, potentially mitigating obesity-related health risks [23,24]. In this context, licorice, widely utilized in traditional Chinese medicine, has garnered attention for its therapeutic benefits [25,26]. Licorice contains ISL, a bioactive chalcone recognized for its antioxidant, anti-inflammatory, and anticancer properties [11]. The integrative approach of utilizing compounds such as ISL from licorice may offer promising avenues for enhancing BAT activity and supporting weight management efforts. To date, the potential of ISL to alleviate HFD-induced obesity in mice by activating BAT remains unexplored.

This study employed gavage administration of ISL to HFD mice to investigate its effects on the activity and function of BAT. The results indicated that, in comparison to the control group, ISL treatment significantly reduced the body weight of HFD mice. This finding is consistent with previous research demonstrating that ISL treatment can attenuate weight gain induced by HFD [14,17,27]. Furthermore, existing studies have shown that extracts of licorice active components can decrease the weight of various adipose depots and the size of adipocytes in the body [28]. Specifically, ISL treatment has been found to significantly lower the weight of mesenteric and perirenal adipose tissues in HFD mice [29]. In line with these observations, our study also noted a significant decrease in the adipose weight-to-body weight ratio in HFD mice following ISL treatment, suggesting that the reduction in body weight may be attributed to decreased lipid deposition in adipose, likely due to the effects of ISL treatment. Moreover, related research indicates that numerous active components from natural medicines can significantly suppress the expression of key adipogenic genes in adipocytes [28,30,31,32], thereby potentially preventing obesity. In this study, ISL treatment led to a significant downregulation of adipogenic genes, accompanied by a reduction in the volume of BAT adipocytes and an increase in the number of small, multifocal lipid droplets. These findings further substantiate the substantial role of ISL, as an extract of licorice active components, in inhibiting lipid deposition.

Numerous studies have indicated that natural flavonoid compounds facilitate the browning of white adipose tissue (WAT) and the thermogenic activity of brown adipose tissue (BAT), thereby modulating lipid metabolism and exerting anti-obesity effects [33,34]. Mitochondria serve as the sole site for UCP1-mediated uncoupling, and any perturbation in mitochondrial function exerts a profound influence on BAT thermogenesis [35]. Notably, ISL has been observed to play a pivotal role in promoting mitochondrial biogenesis and enhancing BAT thermogenesis [34,36]. Consistent with these findings, our study also observed that ISL treatment significantly enhanced the expression of UCP1, UCP2, and other thermogenic genes in the BAT of HFD mice. Additionally, in vitro experiments using C3H10T1/2 cells further validated the promoting effect of ISL on thermogenesis in brown adipocytes. Moreover, ISL has been shown to influence mitochondrial function and biogenesis, with PGC1α appearing to be a key regulatory factor in these processes [37,38,39]. In our study, ISL treatment significantly increased the expression of PGC1α in BAT, thereby confirming its role in promoting mitochondrial biogenesis in BAT. Additionally, pathway enrichment analysis of differentially expressed genes revealed the significant activation of pathways such as the TCA cycle, oxidative phosphorylation, and fatty acid degradation, highlighting the potential impact of ISL on thermogenic and metabolic pathways. In summary, this study provides evidence that ISL treatment can effectively reduce body weight and adipose tissue mass in HFD-induced obese mice, likely through promoting thermogenesis and mitochondrial biogenesis in BAT. These effects are associated with the upregulation of thermogenic genes and PGC1α expression in BAT. Furthermore, our findings are consistent with observations that ISL increases cell viability by reducing apoptosis and oxidative stress through the activation of the Nrf2/HO-1 pathway. These insights contribute to a deeper understanding of the multifaceted benefits of ISL in various biological contexts, including obesity and metabolic disorders [40]. Therefore, ISL may serve as a potential therapeutic agent in the prevention and treatment of obesity and related metabolic disorders.

Cold stimulation has been shown to reduce NNAT expression in adipose tissue, and NNAT knockout mice exhibit significantly enhanced thermogenic capacity [41]. The overexpression of *NNAT* promotes the adipogenic differentiation of 3T3-L1 cells [42], and *NNAT* expression in obese mice is significantly negatively correlated with classical metabolic pathways such as oxidative phosphorylation [43]. These findings suggest that *NNAT* expression promotes lipid deposition and suppresses thermogenesis in adipose tissue. In the present study, ISL treatment significantly decreased *NNAT* expression in the BAT of HFD mice, promoting BAT thermogenesis. This finding is consistent with previous studies and suggests that ISL regulates lipid deposition and thermogenesis in BAT by modulating *NNAT* expression. *SGK1* exhibits a strong correlation with obesity-associated inflammation [44], and its overexpression is associated with adipocyte dysfunction, potentially contributing to the development of obesity, diabetes, and metabolic syndrome [45]. As an epigenetic regulatory factor, *SGK1* promotes the differentiation and lipid deposition of bovine preadipocytes by modulating the phosphorylation and the expression of FOXO1, thereby improving meat quality [46]. In this study, ISL treatment significantly reduced *SGK1* expression in the BAT of HFD-fed mice and suppressed the expression of its differentiation-related genes, aligning with previous findings. However, the precise mechanisms by which ISL regulates genes such as *NNAT* and *SGK1* to influence thermogenesis in BAT remain unclear. Further investigation is required to uncover the detailed molecular pathways and interactions involved in this process. Gaining a deeper understanding of these mechanisms could shed light on the regulation of energy expenditure and provide potential strategies for modulating metabolic health.

## 4. Methods and Materials

### 4.1. Animal Experiment

Six-week-old male C57BL/6 mice were acquired from Hunan SJA Laboratory Animal Co., Ltd. (Changsha, China; approval number SCXK-2019-004). All mice were maintained under controlled conditions, under a 12 h light/dark cycle and 50% relative humidity conditions. They had free access to food and water throughout the rearing period unless otherwise stated. After one week of acclimation on commercial chow, the mice were weighed and randomly assigned to two groups. Twelve mice were placed on an HFD and administered ISL (50 mg/kg body weight daily; Macklin, Shanghai, China, I811911) or saline via gavage. After 8 weeks, the mice were weighed again and euthanized following an overnight fast. The interscapular BAT was extracted, weighed, and stored at −80 °C.

### 4.2. C3H10-T1/2 Cells Differentiation and Oil Red O Staining

C3H10-T1/2 cells were obtained from Procell Life Science and Technology Co., Ltd. (Wuhan, China) and cultured in DMEM/F12 medium (Gibco, Thermo Fisher Scientific, Waltham, MA, USA, 8123199) supplemented with 10% fetal bovine serum (FBS, Gibco, A3161002C). To induce differentiation, 2-day post-confluent C3H10-T1/2 cells (designated as day 0) were incubated in DMEM containing 10% FBS, 1% penicillin/streptomycin, 0.5 mM IBMX (Sigma, Burlington, MA, USA), 10 µg/mL of insulin (Beyotime, Shanghai, China), 1 µM 3-isobutyl-1-methylxanthine (Sigma, MA, USA), 1 nM Triiodothyronine (Sigma, MA, USA), 1 µM Rosiglitazone (Sigma, MA, USA), and 1 µM dexamethasone (Sigma, MA, USA) for 2 days. To sustain differentiation, cells were maintained in DMEM with 10% FBS (Gemini, Calabasas, CA, USA), 1 nM Triiodothyronine, and 10 µg/mL of insulin for the rest of the culture. Unless otherwise stated, ISL (20 μM) was added at the indicated times in some experiments. Following an additional 8 days of culture, lipid droplets in the culture dishes were stained using oil red O solution (Sigma, MA, USA). Subsequently, 200 µL of isopropanol was employed to dissolve the oil red O dye, and the absorbance was measured at 510 nm.

### 4.3. Cell Viability Assay

C3H10-T1/2 cells were seeded in 96-well plates and incubated until confluent. Then, confluent C3H10-T1/2 cells were treated with Cell Counting Kit-8 (Beyotime, Shanghai, China) for 3 h. Absorbance measurements were taken with a microplate reader, and cell viability was determined following the instructions provided by the manufacturer.

### 4.4. Hematoxylin–Eosin (H&E) Staining

The BAT was processed according to standard procedures [47], including fixation with a specialized fat fixative (Servicebio, Wuhan, China, G1119), gradient dehydration, clearing, and paraffin wax infiltration. Using a microtome, the tissue was sectioned into 5 μm slices. These sections were then stained with hematoxylin and eosin and imaged using a fluorescence microscope (Zeiss Axio Observer 3, Oberkochen, Germany).

### 4.5. Immunohistochemistry

The BAT samples were fixed in a specialized fat fixative (Servicebio, G1119), followed by gradient dehydration, paraffin embedding, and sectioning. The tissue sections were then blocked and incubated with a primary antibody UCP1 (1:200; Proteintech, Wuhan, China). After three washes, the sections were incubated with a secondary antibody, followed by three additional washes. The sections were subsequently counterstained, dehydrated, cleared, and mounted. Finally, the sections were examined and imaged using an inverted microscope.

### 4.6. RNA Extraction and Quantitative Real-Time PCR

Total RNA was isolated from BAT utilizing TRIzol reagent (ThermoFisher Scientific, Ward Hill, MA, USA), according to the manufacturer’s guidelines [48]. Reverse transcription was performed with 2 μL of RNA using All-In-One 5X RT MasterMix (Abm, Vancouver, BC, Canada, Cat#G592). qPCR was carried out using the BlasTaq 2X qPCR MasterMix (Abm, Cat#G891) on a real-time PCR instrument (Bio-Rad, Hercules, CA, USA). The primers employed for gene expression quantification are enumerated in Supplementary Table S1. Relative alterations in gene expression, normalized against GAPDH as an internal reference, were assessed using the 2^−ΔΔCT^ method.

### 4.7. Western Blotting Analysis

The BAT samples were triturated, and RIPA buffer (CWBIO, CW2333) containing protease inhibitors (CWBIO, CW2200) was added. The BAT samples were then placed on ice for 30 min. Total protein concentrations were quantified using a BCA protein analysis kit (Beyotime, China). Next, 25 μg of each protein was loaded, and the samples were separated using 12% SDS-PAGE (Bio-Rad). The resulting protein bands were transferred to PVDF membranes for Western blotting. The protein bands were transferred to PVDF membranes for Western blotting. The membranes were then blocked with 5% skimmed milk for 1.5 h at room temperature. The following primary antibodies were used: C/EBPa (Proteintech, 18311-1-AP, 1:2000), FASN (Proteintech, 10624-2-AP, 1:5000), GLUT4 (Santa Cruz Biotechnology, Dallas, TX, USA, sc-53566, 1:2000), PPARG (Proteintech, 16643-1-AP, 1:2000), FABP4 (Proteintech, 12802-1-AP, 1:6000), ATGL (Proteintech, 55190-1-AP, 1:2000), PPARGC1a (Proteintech, 66369-1-Ig, 1:6000), CKB (Proteintech, 15137-1-AP, 1:5000), UCP1 (Proteintech, 23673-1-AP, 1:2000), and GAPDH (Proteintech, 10494-1-AP, 1:5000). The primary antibodies were incubated at 4 °C overnight. The membranes were then washed with TBS with Triton (TBST) and incubated with the HRP Goat Anti-Mouse (Beyotime, A0216, 1:2000) and HRP Goat Anti-Rabbit (Beyotime, A0208, 1:2000) at room temperature for 2 h. Finally, the bound antibody was detected using the Immobilon Western Chemiluminescent HRP substrate (Millipore, Shanghai, China, WBKLS0500) on a chemiluminescence imager (Bio-Rad).

### 4.8. RNA-Seq and Data Analysis

Total RNA was extracted and used to construct cDNA libraries, which were subsequently sequenced on an Illumina platform by Genedenovo Biotechnology Co., Ltd. (Guangzhou, China). Differential gene expression analysis in BAT was performed using DESeq2 software (https://bioconductor.org/packages/release/bioc/html/DESeq2.html, accessed on 15 January 2025). The differentially expressed mRNAs with a *p*-value  <  0.05 and a fold change greater than 1.5 or less than 1.5 were considered significant. Gene Ontology (GO) analysis categorized the DEGs into Biological Process, Cellular Component, and Molecular Function categories. KEGG pathway analysis was conducted using KOBAS software (http://kobas.cbi.pku.edu.cn/, accessed on 15 January 2025), with the FDR-corrected *p*-value threshold set at 0.05.

### 4.9. Statistical Analysis

Data were analyzed using SPSS 23.0 software and presented as mean ± standard deviation (SD). Statistical significance was assessed via *t*-tests, with *p* < 0.05 indicating a statistically significant difference.

## 5. Conclusions

Our study demonstrates that ISL significantly attenuates HFD-induced obesity in mice by increasing the expression of thermogenesis-related genes and proteins in BAT. Key metabolic pathways, including the TCA cycle, oxidative phosphorylation, and fatty acid degradation, are modulated by ISL. Notably, gene expression analysis reveals that ISL downregulates the expression of *NNAT* and *SGK1* in BAT, implicating these genes as potential molecular targets mediating ISL’s effects on BAT function. Collectively, these findings highlight the therapeutic potential of ISL in managing obesity and related metabolic disorders, offering a foundation for further investigation into its molecular mechanisms.

## Data Availability

The data analyzed during the current study are available from the corresponding author on reasonable request.

## Supplementary Materials

The following supporting information can be downloaded at: https://www.mdpi.com/article/10.3390/ijms26041616/s1.
