# Supplementary material for: Isoliquiritigenin Ameliorates High-Fat Diet-Induced Obesity in Mice by Activating Brown Adipose Tissue
Source: Int J Mol Sci. 2025 Feb 14;26(4):1616. doi: 10.3390/ijms26041616 (PMC11855605; doi:10.3390/ijms26041616)
Supplement: Supplementary file 1 [file ijms-26-01616-s001.zip › ijms-3456036-supplementary.pdf]

Table S1. Primers used for qPCR.

Gene name	Primer sequence（5'→3'）
UCP1	F: ACTGCCACACCTCCAGTCATT<br>R: CTTTGCCTCACTCAGGATTGG
NNAT	F: ATCGGCTGGTACATCTTCCG<br>R: TGAACACCTCACTTCTCGCA
Prdm16	F: CCACAAGTCCTACACGCAGT<br>R: GAGGGAGGAGGTAGTGCTGA
Fabp4	F: CGACAGGAAGGTGAAGAGCATCATA<br>R: CATAAACTCTTGTGGAAGTCACGCCT
Cebpa	F: TGCGCAAGAGCCGAGATAAAG<br>R: TCACGGCTCAGCTGTTCCAC
PPARGC1a	F: AGCCGTGACCACTGACAACGAG<br>R: GCTGCATGGTTCTGAGTGCTAAG
ADIPOQ	F: GTCTGTACGATTGTCAGTGGATCTG<br>R: AAGAGGAACAGGAGAGCTTGCA
PPARG	F: CCAAGAATACCAAAGTGCGATCA<br>R: CCAAGAATACCAAAGTGCGATCA
NNAT	F: ATCGGCTGGTACATCTTCCG<br>R: TGAACACCTCACTTCTCGCA
CCDC80	F: GGTGGATGATGATGACTTGGTG<br>R: GGCACTTCGTAGTATTGCTTGACC
IMPHD1	F: GAGTACGCCCGACGTTTTG<br>R: TCTTCAGCCTCACCCCATCT
RARRES2	F: ATTTAAGCTCCAGCAGACCAAC<br>R: CCGGCCTAGAATTTTACCCT
PBLD1	F: CGCTTTGGACTGAGATGGTTT<br>R:AGGGTGCTGTTCCTGTTCTGTAT
SGK1	F: CAGACCGCTGACAAGCTCTACT<br>R: CCCAGGGCACTGGCTATTT
UCP2	F: CTCTACGACTCTGTCAAACAGT<br>R: GGACCTTTACCACATCTGTAGG
GAPDH	F: ATCACTGCCACCCAGAAGACT<br>R: CATGCCAGTGAGCTTCCCGTT
